# Pulmonary Fibrosis and Pulmonary Function in Patients with Metastatic Testicular Cancer During and after Combination Chemotherapy: The BLEOTOX Study

**DOI:** 10.1016/j.euros.2026.04.004

**Published:** 2026-06-03

**Authors:** Lisa Frey, Salma Grunwald, Christian Ruckes, Gregor Duwe, Kerstin Wohlleber, Lisa J. Frey, Maximilian Haack, Niklas Rölz, Robert Dotzauer, Daniel Korczynski, A. Axel Haferkamp, Maximilian P. Brandt

**Affiliations:** a Department of Urology, University Medical Center Johannes Gutenberg University, Mainz, Germany; b Interdisciplinary Center for Clinical Trials, University Medical Center, Johannes Gutenberg University, Mainz, Germany; c Department of Urology, Hospital Group Southwest, Sindelfingen, Germany; d Department of Pneumology, University Medical Center Johannes Gutenberg University, Mainz, Germany

**Keywords:** BEP (bleomycin, etoposide, and cisplatin), Bleomycin-induced pulmonary toxicity, CT thorax, Lung function, Testicular cancer

## Abstract

**Background and objective:**

Metastatic testicular germ cell tumors (mTGCTs) are treated with combination chemotherapy (Cx) containing bleomycin (BEP [bleomycin, etoposide, and cisplatin]). Bleomycin-induced pulmonary toxicity (BPT) is a serious Cx-associated complication. This study aimed to investigate the association between radiographic changes in computed tomography of the chest (CT-T) and pulmonary function tests (PFTs) during, shortly after, and long term after BEP.

**Methods:**

In this nonrandomized, monocentric cohort study, we included patients with mTGCT who were treated with a minimum of two cycles of BEP. Pulmonary changes associated with BPT were extracted from CT-T scans performed before the first (baseline CT-1), after the second cycle (CT-2), and after the last cycle (CT-3) of BEP. Corresponding PFTs were performed before each new cycle. A current PFT was used to evaluate long-term toxicity. Statistical analyses were carried out using the McNemar test, the Fisher exact test, the Wilcoxon signed-rank test, and multivariable regression analyses.

**Key findings and limitations:**

We identified 85 patients (38 had seminomas, and 47 had nonseminomas). Thirty-two patients received a current PFT. After two cycles of BEP, 21% of the patients showed radiographic BPT and 20% PFT deterioration. The association between CT and PFT changes was significant (odds ratio [OR] = 10.65 [95% confidence interval {CI} = 3.1–37.6]; *p* < 0.001). During BEP, the proportion of patients with deterioration in PFTs increased from 7.1% to 22% (Δ = 0.176 [95% CI = 0.076 – 0.277]; *p* = 0.0046). All patients with current PFT deterioration showed BPT after the second BEP cycle (CT-2); however, the association was not significant (OR = 6.25 [95% CI = 0.68–57.9]; *p* = 0.14).

**Conclusions and clinical implications:**

BPT is a common radiographic finding during and after BEP. Radiographic changes in CT-2 were significantly associated with reduced PFT. However, in most patients, PFT recovered over time with no radiographic correlation to any time point of CT-T during BEP. Therefore, routine CT-T during ongoing BEP should not be performed for BPT detection and should only be considered in patients who are clinically symptomatic.

## Take Home Message

Bleomycin-associated toxicity (BPT) with radiographic pulmonary changes is a common finding during bleomycin, etoposide, and cisplatin combination therapy, and there is a significant association with reduced pulmonary function. Most patients recover; nevertheless, close monitoring with pulmonary function tests and computed tomography of the thorax can be essential to prevent BPT.

ADVANCING PRACTICE **What does this study add?**

Bleomycin-induced pulmonary toxicity (BPT) is a common finding in computed tomography of the thorax (CT-T) after two cycles of combination chemotherapy (Cx). BPT is associated significantly with reduced pulmonary function tests (PFTs). Interestingly, all patients with reduced PFTs during long-term follow-up showed bleomycin-associated pulmonary changes on CT-T. Therefore, routine CT-T during Cx might aid clinicians in identifying patients at risk for long-term deterioration of their pulmonary function.

**Clinical Relevance**

This study suggests that bleomycin-associated pulmonary changes on chest CT are common during BEP chemotherapy for metastatic testicular cancer and correlate with contemporaneous pulmonary function deterioration, although most patients recover over time. These findings support symptom-driven imaging and serial pulmonary function monitoring, while highlighting the need to identify patients at risk of persistent long-term toxicity. Associate Editor: Maria Carmen Mir

**Patient Summary**

Metastatic testicular germ cell tumors are typically treated with chemotherapy, which may lead to life-threatening pulmonary alterations. We found that pulmonary changes were frequently observed in computed tomography of the chest with decreased lung function after two cycles of chemotherapy. Most patients, but not all, recovered from reduced pulmonary function. However, those patients who had long-term changes in pulmonary function also had changes during chemotherapy on CT. Therefore, CT scans of the chest might help identify patients at risk of long-term pulmonary dysfunction.

## 1 Introduction

Metastatic testicular germ cell tumors (mTGCTs) are highly curable, with an overall cure rate exceeding 90% [1]. According to the International Germ Cell Cancer Cooperative Group (IGCCCG) classification, patients with metastatic disease are treated with one to four cycles of combination chemotherapy (Cx) consisting of bleomycin, etoposide, and cisplatin (BEP). Despite excellent cure rates, increased morbidity during BEP Cx is of pivotal clinical relevance [2]. In this context, bleomycin-induced pulmonary toxicity (BPT) represents one of the most feared adverse side effects of bleomycin, which can cause an interstitial pneumonitis and potentially lethal pulmonary fibrosis [3], [4]. The pathogenesis of BPT is not entirely understood, but oxidative damage, damage by cytokines, and deficiency of bleomycin-metabolizing enzymes seem to play a critical role in the development of BPT [5], [6]. Previous studies reported several risk factors for the development of BPT, including high cumulative bleomycin dose, smoking, reduced renal function, and being aged >40 yr [4], [7].

Currently, there are no standardized protocols for monitoring BPT during BEP, and there is no consensus statement in international guidelines on the frequency of pulmonary function tests (PFTs) or computed tomography of the thorax (CT-T) [1], [8]. Nevertheless, repetitive PFTs or CT-T are recommended in case of symptoms such as dry cough, dyspnea, and crackles during auscultation [9], [10], [11]. BPT changes on CT-T are a common finding, with a reported incidence of ≈70% on restaging CT-T [7]. However, no data exist on whether patients with radiographic changes on CT-T during BEP might be at higher risk of long-term pulmonary function deterioration.

This study aimed to investigate the prevalence of CT-T changes during BEP and the correlation of CT-T changes with respective PFTs of patients during and after BEP. Furthermore, we aimed to analyze the association of BPT-associated changes during initial BEP and long-term pulmonary changes measured with a current PFT.

## 2 Patients and methods

This is a retrospective, nonrandomized cohort study including patients with mTGCT who were treated with one to four cycles of BEP at our department between 2014 and 2023. Treatment interval was 21 d with bleomycin 30 mg on d 1, 8, and 15; etoposide 100 mg/m^2^; and cisplatin 20 mg/m^2^ on d 1–5. Decisions regarding modification of therapy, including whether to continue or discontinue bleomycin, are highly individualized and made in an interdisciplinary setting.

To assess long-term toxicity, all patients were recontacted. Thirty-two patients provided written informed consent for a current PFT and tumor marker control and were included in a prospective cohort. For the final analysis, we included only patients with two or more cycles of BEP, assuming that one cycle of BEP with 90 mg bleomycin would not result in PFT deterioration [4], [12]. After two cycles of BEP, CT-T (CT-2) and PFT (PFT-2) were both performed within a median of 5 d (interquartile range [IQR] = 0–6); this time point was the primary choice for investigating the association between changes in CT-T and PFT. Patient characteristics and risk factors for BPT (age; smoking; renal function; tumor markers: α-fetoprotein [AFP], β-human chorionic gonadotropin, and lactate dehydrogenase) were extracted from digital patient data files. This study was approved by the local ethics committee (protocol number: 2021-16037).

### 2.1 Radiographic assessment

Radiographic assessment before BEP, after the first two cycles of BEP, and after completion of BEP included a CT-T and a CT of the abdomen or a CT-T and a magnetic resonance imaging of the abdomen. CT-T scans are defined as CT-1 (baseline) before starting BEP, CT-2 after two cycles, and CT-3 (final) after completing Cx. Radiographic changes were assessed with the corresponding existing radiographic report extracted from digital data files and evaluated for lesions that were associated with BPT (typically bilateral and basal-peripheral; early signs: ground-glass opacities, septal thickening, reticular changes; late signs: fibrosis with honeycombing and traction bronchiectasis) [9], [13]. Pulmonary associated with BPT were documented separately. CT-T scans were performed according to standard protocol (in a caudal-to-cranial direction from the costophrenic pleural recesses to above the thorax aperture) using a native technique.

### 2.2 PFTs

Patients received spirometry before treatment initiation (PFT baseline) and before each subsequent cycle of BEP Cx: PFT-1 (before cycle 2), PFT-2 (before cycle 3), and PFT-3 (before cycle 4/after cycle 3). An additional PFT was performed when there were previous abnormalities or the patient reported clinical symptoms. Extracted data from PFTs were total lung capacity (TLC), vital capacity (VC), and forced expiratory volume in one second (FEV1) as well as diffusing capacity for carbon monoxide (DLCO) whenever available. Since pulmonary fibrosis is associated with restrictive pulmonary function, our pulmonologist considered a decrease in TLC of >10%, a FEV1 of <75%, and/or a reduction of VC <60% of the normal values and a progressive decline in TLC and VC over time as suggestive of a clinically relevant restrictive ventilation disorder [14], [15], [16]. All PFTs were evaluated by a pulmonologist, allowing reliable identification of a restrictive ventilation disorder suggestive of bleomycin-associated pulmonary toxicity during or after therapy. For the assessment of long-term pulmonary function, a follow-up PFT was performed in all patients who agreed to participate, with a minimum interval of ≥6 mo after the last cycle of BEP. Patients with obstructive ventilation disorders on PFT or previously known pulmonary dysfunctions were excluded.

### 2.3 Statistical analysis and ethical approval

Descriptive statistics were reported as frequencies and proportions. Continuous variables were presented as medians ± IQR or means ± standard deviations. The McNemar test was used for paired categorical variables (PFT changes over time), with calculation of the treatment difference (Δ). The Fisher exact test compared categorical variables between testing methods (PFT-CT changes), with effect sizes expressed as odds ratios (ORs). The Wilcoxon signed-rank test was applied for nonnormally distributed paired samples together with the standardized mean differences (Cohen d). All effect estimates are reported with 95% confidence intervals (CIs). Findings suggestive of BPT (restrictive ventilatory impairment on PFT or typical imaging findings indicative of pneumonitis on CT) were coded positive or negative for BPT-associated changes. We performed a multivariable binary logistic regression analysis based on the data from the retrospective study cohort. Therefore, we pooled the occurrence of BPT-associated changes reported in PFT 1–3 and CT-2-3. The multivariable model was adjusted for known risk factors for BPT or risk factors associated with impaired pulmonary function (being aged ≥40 yr, having pulmonary alterations including asthma and pulmonary metastases, having elevated creatine and BEP cycles, and obesity). Results with p < 0.05 were considered statistically significant. All data were analyzed and visualized using IBM SPSS Statistics (version 27; IBM Corp., Armonk, NY) and GraphPad Prism (version 10; GraphPad Software, San Diego, CA).

## 3 Results

### 3.1 Baseline patient characteristics

**Fig. 1:**
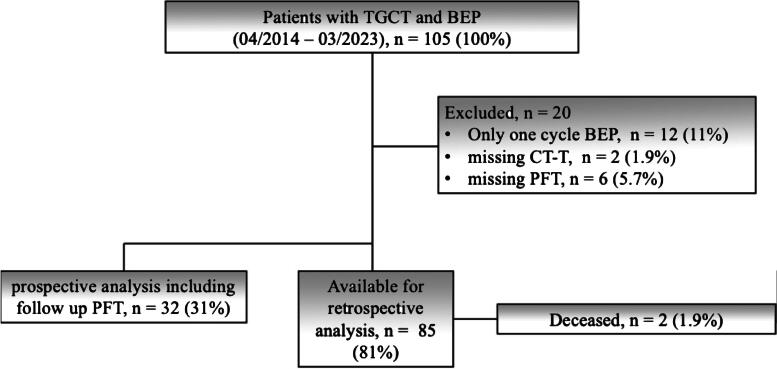
CONSORT diagram of patients included in the BLEOTOX study. TGCT = testicular germ cell tumors; BEP = bleomycin, etoposide, and cisplatin; CT-T = computed tomography of the thorax; PFT = pulmonary function test; CONSORT = Consolidated Standards of Reporting Trials;.

Overall, 105 patients were identified who received BEP. Twenty patients were excluded if they received fewer than two cycles or had incomplete data (Fig. 1). Thirty-two patients provided consent to follow-up PFT. Two patients died because of pulmonary complications during BEP. In addition to the two fatal cases, three additional patients developed clinically symptomatic pneumonitis.

Baseline characteristics of the study population are summarized in Table 1. Overall, 85 men were treated with two to four cycles of BEP according to their risk classification. The median age at diagnosis was 34 yr; 38 patients had a seminoma, and 47 patients had a nonseminoma. Most patients (89%, *n* = 76) had a good prognosis according to IGCCCG and received three cycles of BEP (80%, *n* = 68). In the multivariable regression analysis, patients aged >40 yr were at increased risk for abnormalities on CT-2-3 (OR = 3.66 [95% CI = 1.19–11.27]; *p* = 0.024), whereas no significant increase in PFT abnormalities was observed (OR = 0.41 [95% CI = 0.11–1.52]; *p* = 0.2). No statistical significance was observed for any of the other known risk factors for BPT (Table 2).

**Table 1:** Patient characteristics of the retrospective and follow-up cohorts

Characteristic		Retrospective cohort	Follow-up cohort
Total patients, *n* (%)		85 (100)	32 (100)
Age (yr), median (IQR)		34 (28–46)	37 (28–48)
BMI kg/m^2^, median (IQR)		27.17 (24–30)	27.6 (24.4–27.8)
**Histopathology, *n* (%)**			
	Nonseminoma	47 (55)	17 (53)
	Seminoma	38 (45)	15 (47)
**IGCCCG prognosis group, *n* (%)**			
	Good	76 (89)	29 (91)
	Intermediate	3 (3.5)	1 (3)
	Poor	6 (7.1)	2 (6)
**Clinical stage, *n* (%)**			
	I	12 (14)	6 (19)
	II	58 (68)	21 (66)
	III	15 (18)	5 (15)
**BEP cycles, *n* (%)**			
	2	7 (8.2)	1 (3)
	3	68 (80.0)	27 (84)
	4	10 (12)	4 (13)
**Smoking, *n* (%)**			
	Current	25 (29)	9 (28)
	Former	4 (4.7)	1 (3)
	Never	56 (66)	22 (69)
Asthma, *n* (%)		6 (7)	2 (6.3)
Pulmonary metastases, *n* (%)		9 (11)	3 (9.4)
Clinical symptoms of pneumonitis, *n* (%)		5 (5.9)	2 (6.3)
**Creatinine level, *n* (%)**			
	Normal	81 (95)	31 (97)
	Elevated	4 (4.7)	1(3)

IQR = interquartile range; BMI = body mass index; IGCCCG = International Germ Cell Cancer Collaborative Group; BEP = bleomycin, etoposide, and cisplatin.

**Table 2:** Statistical analysis of known risk factors for BPT and the occurrence of abnormalities in PFT and CT of the thorax imaging

Factor	Restrictive ventilation disorder, (PFT 1–3)			BPT-associated changes, (CT-2-3)		
	OR	95% CI	*p* value	OR	95% CI	*p* value
Aged ≥40 yr	0.41	0.11–1.52	0.2	3.66^a^	1.19–11.27^a^	0.024^a^
Asthma	2.52	0.35–18.39	0.4	0.38	0.05–2.69	0.3
Obesity	1.58	0.44–5.67	0.5	0.79	0.27–2.29	0.7
Elevated creatinine	1.73	0.12–24.94	0.7	0.38	0.04–3.92	0.4
Pulmonary metastasis	1.72	0.21–13.9	0.6	0.83	0.13–5.18	0.8
BEP cycles	0.65	0.14–2.96	0.6	1.46	0.39–5.46	0.6

BEP = bleomycin, etoposide, and cisplatin; BPT = bleomycin-induced pulmonary toxicity; CI = confidence interval; CT= computed tomography; CT-2 = after two cycles of BEP; CT-3 = final staging 6 weeks after completion of BEP; OR = odds ratio; PFT = pulmonary function test; PFT-1 to PFT-3 = after one/two/three cycles of BEP

a Multivariable binary logistic regression analysis was performed based on the retrospective study cohort (*n* = 85, BEP cycles coded as 2–4).

### 3.2 Radiographic changes and PFT parameters during chemotherapy

**Fig. 2:**
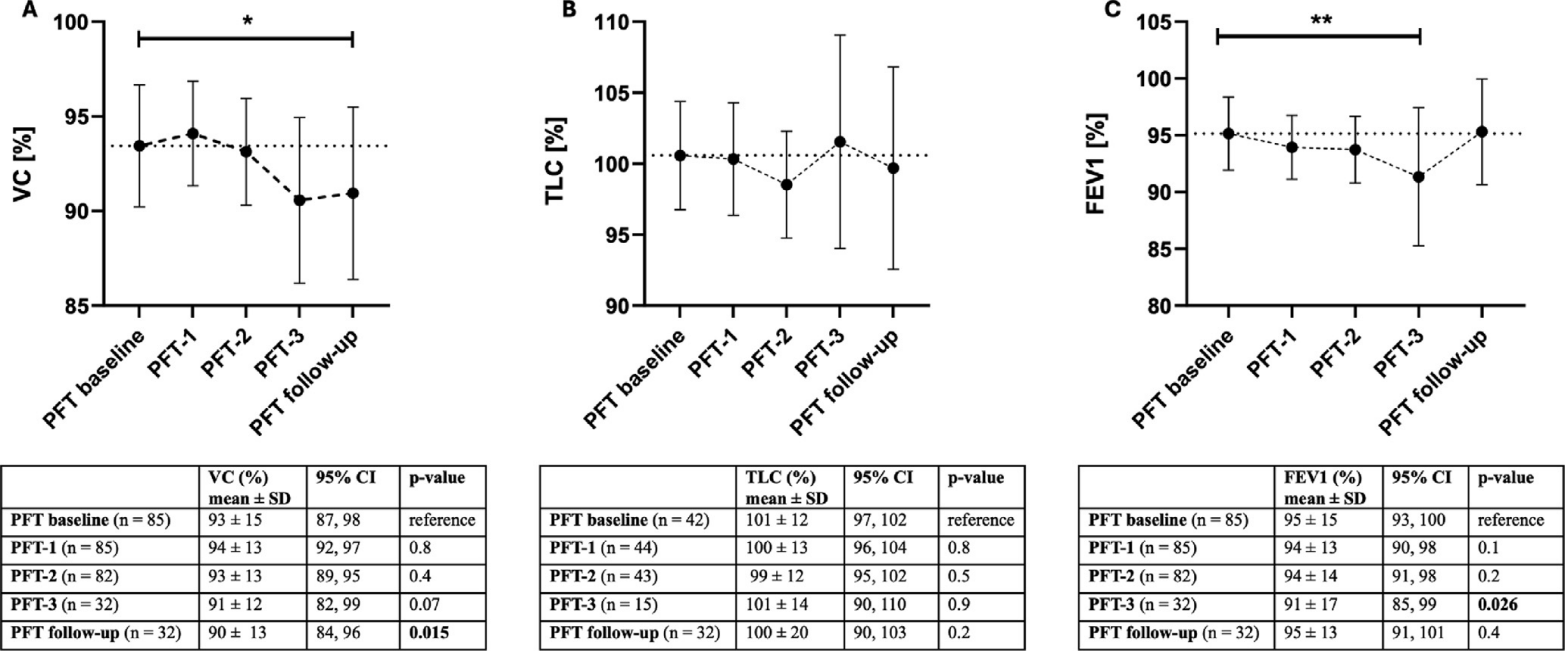
Mean pulmonary function parameters (95% CI) at baseline, during treatment, and at long-term follow-up. (A) VC tended to decrease with no statistical significance but showed a persisting deterioration at long-term follow-up. (B) TLC with no statistically significant differences between time points. (C) Reduced FEV1 declined significantly over time (PFT baseline to PFT-3. Cx = combination chemotherapy; PFT baseline = PFT before Cx; PFT-1 to PFT-3 = PFT after one to three cycles of Cx; PFT–follow-up = current PFT after completion of Cx; CI = confidence interval; VC = vital capacity; TLC = total lung capacity; FEV1 = forced expiratory volume in one second; PFT = pulmonary function test. *p = 0.015. **p = 0.026.

After two cycles of BEP, CT-2 was available for 84 patients (missing n = 1). Radiographic changes were present in 58% of the patients (*n* = 49), with 21% (*n* = 18) classified as fibrotic and defined as BPT. After three cycles, CT-3 was available for 80 patients (missing *n* = 5). BPT-associated changes in CT-3 were found in 51% of the patients (*n* = 41), whereas 25% (*n* = 20) had no pathological results. Of those with BPT-associated changes in CT-2, 15 patients had persistent changes in CT-3. Eleven patients with nonspecific changes on CT-2 showed progression to BPT-associated changes on CT-3. In addition, 15 patients with a completely unremarkable CT-2 developed BPT-associated changes in CT-3 (Table 3). FEV1 declined significantly with increasing bleomycin exposure (PFT baseline to PFT-3: *n* = 32; Cohen d = 0.25; *p* = 0.026). VC tended to decrease over time relative to baseline, but the decline did not reach statistical significance (PFT baseline to PFT-3: *n* = 32; Cohen d = 0.2; *p* = 0.07). TLC remained stable throughout the entire study period (PFT baseline to PFT-3: *n* = 8; Cohen d = 0.07; *p* = 0.9) (Fig. 2 and Supplementary Table 1). When evaluated by an expert pulmonologist, the proportion of patients with deterioration in PFTs increased from 7.1% at baseline to 22% at PFT-1 (Δ = 0.176 [95% CI = 0.076–0.277]; *p* = 0.0046) and then slightly declined to 20% at PFT-2 (Δ = 0.122 [95% CI = 0.010–0.234]; *p* = 0.033).

**Table 3:** BPT- and non-BPT–associated radiographic changes in CT-T during and after Cx

CT-2	*n* = 84, *n* (%)
BPT-associated changes	18 (21)
Non-BPT–associated changes	31 (37)
No changes	35 (42)

**CT-3**	***n* = 80, *n* (%)**

BPT-associated changes	41 (51)
Non-BPT–associated changes	19 (24)
No changes	20 (25.0)

BPT = bleomycin-induced pulmonary toxicity; Cx = combination chemotherapy; CT-T = computed tomography of the thorax; CT-2 = CT of the thorax after two cycles of Cx; CT-3 = CT of the thorax after three cycles of Cx.

### 3.3 Association of radiographic changes and pulmonary function during BEP

Data for PFT-1 (after one cycle of BEP) were available for 85 patients, whereas PFT-2 (after two cycles of BEP) was available for 82 patients. Paired data for PFT-2 and CT-2 were available in 81 cases. Sixteen patients (20%) showed a restrictive ventilation disorder in PFT-2. Combined BPT-associated changes in CT-2 and PFT-2 occurred in nine patients (11%), whereas a restrictive ventilation disorder with unremarkable or nonspecific CT-T findings was present in seven patients (8.6%). There was a significant association between CT-2 and PFT-2 (OR = 10.65 [95% CI = 3.1–37.6]; *p* < 0.001; Table 4).

**Table 4:** Association between lung function and radiographic changes

		PFT-2	
		Normal, *n* (%)	Reduced, *n* (%)
CT-2	BPT-associated changes	7 (8.6)	9 (11)
	Non-BPT–associated changes	29 (36)	2 (2.5)
	No changes	29 (36)	5 (6.2)

BPT = bleomycin-induced pulmonary toxicity; PFT-2 = pulmonary function test after two cylces of chemotherapy; CT-2 = computed tomography of the thorax after two cycles of chemotherapy.

### 3.4 Correlation of radiographic changes and pulmonary function after BEP chemotherapy

In the prospective cohort, four of the 32 individuals had signs of a restrictive ventilation disorder in lung function. The last cycle of Cx was more than two years ago in all four patients. All of them had BPT-associated findings at CT-2 and/or CT-3, and two patients already had a restrictive ventilation disorder in PFT-2. Of the remaining 28 patients with normal PFT follow-up, 10 patients had a restrictive ventilation disorder in their previous PFTs, and 11 patients had BPT-associated changes in CT-3. Both findings occurred in five individuals. At follow-up, FEV1 recovered (no significant difference between PFT baseline and PFT follow-up: *n* = 32; Cohen d = 0.007; *p* = 0.4), whereas VC remained reduced (PFT baseline to PFT follow-up: *n* = 32; Cohen d = 0.18; *p* = 0.015) (Fig. 2 and Supplementary Table 1). The probability of a normal PFT follow-up with a previously unremarkable CT-2 and CT-3 was 92% and 93%, respectively. A potential trend was observed; however, neither the association between BPT-associated changes in CT-2 and PFT follow-up (OR = 6.25 [95% CI = 0.68–57.9]; *p* = 0.14) nor the association between BPT-associated changes in CT-3 and PFT follow-up (OR = 10 [95% 0.47–214]; *p* = 0.09) reached statistical significance.

### 3.5 Oncologic follow-up

In the prospective cohort (*n* = 32), no TGCT recurrences occurred, and all patients were alive at data acquisition. Tumor markers were available for 31 patients across key time points. Two patients showed mildly elevated AFP (maximum 11 ng/mL, normal AFP <8.8 ng/mL), not linked to recurrence.

During the initial treatment period, two patients (aged >50 yr, stage II, and good prognosis) died because of BPT. Except for age, they had no other risk factors, such as impaired kidney function and asthma, and were nonsmokers. BPT-associated changes were present in CT-2; however, only one patient had a suspicious PFT-2. The other three patients with clinically symptomatic pneumonitis (aged ≥40 yr, one with mild renal impairment) had a restrictive ventilation disorder on PFT-2 and BPT-associated changes on CT-2.

## 4 Discussion

To our knowledge, this is the first study that evaluates radiographic changes measured by CT-T in correlation with PFTs for patients with mTGCT who received at least two cycles of BEP. With ≈20% (after two cycles of BEP) and >50% (after three cycles of BEP), BPT-associated changes are a common finding on CT-T, and treatment continuation must be weighed against the risk of fulminant pneumonitis. However, routine imaging during BEP for the purpose of detecting BPT should not be considered in patients who are asymptomatic but rather in patients with symptoms. Still, CT findings may be frequent, but they do not imply clinically relevant pneumonitis. Additionally, it should be emphasized that the clinical diagnosis of BPT should not only be guided by imaging modalities. Clinical symptoms and repetitive PFTs before each new cycle must be carefully taken into consideration. Comparison between studies is challenging because of the lack of consensus for the definition of BPT and how to monitor these patients during BEP. There are a few studies that used imaging for the definition of fibrotic changes on CT-T or chest x-ray [4] and others that focused on restrictive pulmonary function results [10] or the presence of clinical symptoms [4]. Clear criteria to identify clinically relevant pneumonitis, based on radiographic, clinical, or PFT changes, are lacking. Consequently, the physicians must rely on clinical judgment and individual experience to guide management decisions.

Notably, in our cohort, two patients whose only risk factor was being aged >40 yr died from BPT. Both patients had no pulmonary symptoms, whereas the second patient presented with suspicious findings in PFT-2 and CT-2. Consequently, for the second patient, a repeat PFT was conducted, and after thorough interdisciplinary consultation with the pulmonologist, a decision was made to continue the BEP regimen. These two clinical scenarios highlight two important aspects. First, in both cases, nearly the maximum cumulative dose of bleomycin (270 mg for the first patient and 240 mg for the second) was administered, demonstrating the association between increased dosing and toxicity. Second, both cases exemplify clinical situations in which the patient’s deterioration occurred so rapidly that it could not be prevented despite exhaustive efforts. Apart from the two patients who died, three patients (aged 40, 54, and 55 yr) in our cohort developed clinically symptomatic pneumonitis. All three exhibited a restrictive ventilation disorder in PFT-2 and BPT-associated changes on CT-2. Nevertheless, only one patient developed clinical symptoms after two cycles of BEP, prompting a switch to VIP, whereas the other two became symptomatic only shortly after, or at the end of, the final cycle (with the last bleomycin dose omitted). Despite marked functional and radiographic BPT-associated changes, clinical symptoms were mild in all three patients. Notably, five patients (three aged >40 yr) with signs of restrictive ventilation disorder on PFT-2 and BPT-associated changes on CT-2 did not develop any clinical symptoms of pneumonitis, underscoring the inherent challenge in defining parameters capable of predicting BPT. A significant association between BPT-associated changes in CT-2 and PFT-2 should prompt closer monitoring of patients with imaging and functional changes—particularly when both are present in a single patient—and even mild symptoms should trigger interdisciplinary discussion regarding potential therapy modification. Furthermore, all patients with clinically manifest pneumonitis were aged >40 yr. In concordance with other studies, this suggests that patient age should be carefully considered, and alternatives to bleomycin should be discussed in older patients [4], [7].

However, in PFT, restrictive ventilation disorders can be a first sign of pneumonitis. According to the literature, a relevant restrictive ventilation disorder was assumed if TLC was reduced, the FEV1 was restricted, and/or a continued reduction in VC was present [10], [17], [18]. The usefulness of PFTs, even in the absence of clinical symptoms, is also subject to controversial debate. Some investigators do not recommend the implementation of PFT in the absence of clinical symptoms to rule out the risk of reduced oncological efficacy [19]. In clinical practice, many confounding factors, such as patient compliance during PFT, examination conditions, and intraobserver variability, exist that potentially influence the quality of the respective PFT. More importantly, the diagnosis of impaired PFT must be interpreted in the context of earlier PFTs or must at least be interpreted carefully depending on the results and patient compliance itself. Therefore, analyses of sequential PFTs might be more relevant than a single PFT to confirm or rule out impaired pulmonary function. Still, some parameters such as the DLCO, VC, and TLC might detect lung damage before it is radiographically apparent [17], [18], [20], [21]. In contrast, the practical limitation of DLCO is that special diagnostic equipment is necessary, which is not ubiquitously available and has not been analyzed in our cohort for every patient. Bellamy et al [17] found a lower VC and TLC in patients with moderate and severe lung damage on postchemotherapy imaging with chest x-ray and CT-T. In contrast to our analyses, PFTs were only conducted after completion of Cx (a minimum of four cycles of BEP) and not during ongoing therapy.

Comparable to Lauritsen et al [10], we found a nearly complete recovery of PFT with at least six months between completion of Cx and follow-up testing. Therefore, it can be assumed that most of the patients with BPT will recover over time. Despite missing statistical significance in the follow-up cohort, four of the 32 patients, all of whom had BPT-associated changes in CT-2/CT-3, still showed a reduction in lung function more than two years after completion of Cx. We consider this clinically relevant, as no patient with unremarkable CT-2 and CT-3 had impaired PFT in our follow-up cohort. Therefore, patients with changes in CT-2 or CT-3 should be informed about potential long-term pulmonary side effects, and omitting bleomycin must be considered as a reasonable alternative for risk groups compared with full-dose BEP [1], [22]. Analysis of larger cohorts is necessary to verify these results.

In a study by O’Sullivan et al [4], the authors retrospectively reviewed 825 datasets of patients with TGCT who received bleomycin-containing Cx between 1982 and 1999. BPT was present in 6.8% of the patients (57/835). The diagnosis was based on typical radiographic changes (CT-T or x-ray) or clinical symptoms requiring cortisone therapy or when BPT was the primary cause of death [4]. In our cohort, there were more BPT cases diagnosed by CT-T (CT-2: 21% and CT-3: 51%) as compared with the aforementioned study. One possible reason for the higher rate of BPT in our study is that CT-T is more sensitive to pulmonary morphological changes, such as fibrotic remodeling, compared with conventional x-ray. This fact is supported by the work of Bellamy et al [17]. This study group reviewed 100 CT-T scans of patients with TGCT receiving bleomycin and compared these data with the simultaneous chest x-ray images. BPT-associated changes were significantly more often detected on CT-T (38%) compared with conventional x-ray images (15%).

A limitation of our study is the retrospective study design and the limited but expected number of patients. Nevertheless, this dataset was well investigated with complete clinical and imaging data and a large prospective follow-up group (32/85; 38%). A potential selection bias in the follow-up cohort should be acknowledged when interpreting the results. Nevertheless, the follow-up cohort closely resembled our overall cohort in terms of clinical parameters. Although structured follow-up data are not available for all patients, those with relevant pulmonary issues or tumor recurrence would likely have presented to our center. Additionally, the data analyses might be limited by low statistical power because of a small sample size and outcome rarity. Because of the rare occurrence, pooling of the time points was necessary for the multivariable regression analysis, which could potentially affect the statistical conclusions. The definitive decision whether a restrictive change was present or not was ultimately made by an expert pulmonologist (D.K.) and not solely based on absolute values. In our cohort, a significant number of patients showed a persistent decline in VC at PFT follow-up, whereas the significantly reduced FEV1 (after three cycles of BEP) fully recovered to baseline. Although both changes were statistically significant, their effect sizes were small. The available TLC data are insufficient to draw statistically valid conclusions. Comparison of these three parameters indicates that a clinically relevant assessment of existing or persistent restrictive changes should not be based solely on absolute measurements. However, we would like to note that this may complicate the interpretation of the results and limit comparability. Moreover, we did not perform extended spirometry with determination of DLCO for every patient and therefore cannot make any statements about the value of the DLCO for the assessment of BPT and imaging changes.

This is the first study that included patients with a CT-T before, during, and after the completion of BEP, which allows us to analyze sequential high-resolution CT-T with repetitive PFTs.

## 5 Conclusions

With 21% and 51% of the patients after two and three cycles of BEP, respectively, BPT-associated pulmonary changes are a common CT-T finding. In the patient group who received current PFTs, a nearly complete recovery of PFT was observed. Nevertheless, it should be highlighted that four of the 32 individuals had reduced lung function two years after completion of BEP, all of whom had BPT-associated changes at CT-2 and/or CT-3. Close monitoring of lung function with sequential PFT and CT-T in the case of symptoms is necessary to avoid potentially life-threatening BPT.

  ***Author contributions:*** Lisa Frey had full access to all data in the study and takes responsibility for the integrity of the data and the accuracy of the data analysis.

  *Study concept and design:* Frey, Wohlleber, Korczynski, Brandt.

*Acquisition of data:* Frey, Grunwald, Wohlleber, Brandt.

*Analysis and interpretation of data:* Frey, Grunwald, Korczynski, Haack, Brandt.

*Drafting of the manuscript:* Frey, Duwe, Haack, Brandt.

*Critical revision of the manuscript for important intellectual content:* all authors.

*Statistical analysis:* Frey, Ruckes, Brandt.

*Obtaining funding:* None.

*Administrative, technical, or material support:* None.

*Supervision:* Brandt, Haferkamp.

*Other:* None.

  ***Financial disclosures:*** Lisa Frey certifies that all conflicts of interest, including specific financial interests and relationships and affiliations relevant to the subject matter or materials discussed in the manuscript (eg, employment/affiliation, grants or funding, consultancies, honoraria, stock ownership or options, expert testimony, royalties, or patents filed, received, or pending), are the following: None.

  ***Funding/Support and role of the sponsor:*** None.

  ***Acknowledgements:*** The authors wish to thank all patients for their contribution.

  ***Ethical approval:*** All procedures performed in this study involving human participants were in accordance with the ethical standards of the institutional and national research committee and with the 1964 Declaration of Helsinki and its later amendments or comparable ethical standards. This study was approved by the ethics committee of the Rhineland-Palatinate State Medical Association (application number: 2021-16037).

  ***Consent to participate:*** Informed consent was obtained from all individual participants included in this study.

  ***Consent to publish:*** Informed consent was obtained from all participants regarding publishing their data.
