# Supplementary material for: Pulmonary Fibrosis and Pulmonary Function in Patients with Metastatic Testicular Cancer During and after Combination Chemotherapy: The BLEOTOX Study
Source: Eur Urol Open Sci. 2026 Jun 3;89:63–70. doi: 10.1016/j.euros.2026.04.004 (PMC13262170; doi:10.1016/j.euros.2026.04.004)
Supplement: Supplementary Data 1 [file mmc1.docx]

**Supplement 1.** **BPT (bleomycin induced pulmonary toxicity) associated changes over time measured by PFT (pulmonary function test).** PFT-1: PFT after one cycle of chemotherapy (Cx), PFT-2: PFT after two cycle of Cx, PFT-3: PFT after three cycle of Cx, PFT-4: PFT after four cycle of Cx, PFT-5: follow-up after completion of Cx

|  | **PFT-1** | **PFT-2** | **PFT-3** | **PFT-4** | **PFT-5** |
| --- | --- | --- | --- | --- | --- |
| **BPT associated changes** | 6 (7.1%) | 19 (22.4%) | 16 (19.5%) | 5 (15.6%) | 4 (12.4%) |
| **no BPT associated changes** | 79 (92.9%) | 66 (77.6%) | 66 (80.5%) | 27 (84.4%) | 28 (87.5%) |
